# Mode of primary cancer detection as an indicator of screening practice for second primary cancer in cancer survivors: a nationwide survey in Korea

**DOI:** 10.1186/1471-2407-12-557

**Published:** 2012-11-26

**Authors:** Beomseok Suh, Dong Wook Shin, So Young Kim, Jae-Hyun Park, Weon Young Chang, Seung Pyung Lim, Chang-Yeol Yim, Be-Long Cho, Eun-Cheol Park, Jong-Hyock Park

**Affiliations:** 1 Department of Family Medicine & Health Promotion Center, Seoul National University Hospital, Seoul, Republic of Korea; 2 Cancer Survivorship Clinic, Seoul National University Cancer Hospital, 101 Daehak-ro, Jongno-Gu, Seoul, 110-744, Republic of Korea; 3 Division of Cancer Policy and Management, National Cancer Control Institute, National Cancer Center, Goyang, Republic of Korea; 4 Department of Social and Preventive Medicine, Samsung Biomedical Research Institute, Sungkyunkwan University School of Medicine, Suwon, Republic of Korea; 5 Department of General Surgery, Cheju Regional Cancer Center, Cheju National University Hospital, Jeju, Republic of Korea; 6 Department of Thoracic and Cardiovascular Surgery, Daejon Regional Cancer Center, Chungnam National University Hospital, Daejon, Republic of Korea; 7 Division of Hematology-Oncology, Jeonbuk Regional Cancer Center, Chonbuk National University Hospital, Jeonju, Republic of Korea; 8 Department of Preventive Medicine & Institute of Health Services Research, Yonsei University College of Medicine, Seoul, Republic of Korea; 9 Division of Cancer Policy and Management, National Cancer Control Research Institute, National Cancer Center, 111 Jungbalsan-ro, Ilsandong-gu, Goyang-si, Gyeonggi-do, 410-769, Republic of Korea

**Keywords:** Cancer survivor, Second primary cancer, Screening, Mode of detection, Screen-detected

## Abstract

**Background:**

While knowledge and risk perception have been associated with screening for second primary cancer (SPC), there are no clinically useful indicators to identify who is at risk of not being properly screened for SPC. We investigated whether the mode of primary cancer detection (i.e. screen-detected vs. non-screen-detected) is associated with subsequent completion of all appropriate SPC screening in cancer survivors.

**Methods:**

Data were collected from cancer patients treated at the National Cancer Center and nine regional cancer centers across Korea. A total of 512 cancer survivors older than 40, time since diagnosis more than 2 years, and whose first primary cancer was not advanced or metastasized were selected. Multivariate logistic regression was used to examine factors, including mode of primary cancer detection, associated with completion of all appropriate SPC screening according to national cancer screening guidelines.

**Results:**

Being screen-detected for their first primary cancer was found to be significantly associated with completion of all appropriate SPC screening (adjusted odds ratio, 2.13; 95% confidence interval, 1.36–3.33), after controlling for demographic and clinical variables. Screen-detected cancer survivors were significantly more likely to have higher household income, have other comorbidities, and be within 5 years since diagnosis.

**Conclusions:**

The mode of primary cancer detection, a readily available clinical information, can be used as an indicator for screening practice for SPC in cancer survivors. Education about the importance of SPC screening will be helpful particularly for cancer survivors whose primary cancer was not screen-detected.

## Background

With unprecedented innovation in detection, diagnosis, and treatment for cancer over the recent years, the overall survival rate for cancer has significantly increased [1]. As a result, the number of cancer survivors more than tripled from 1970 to 2000, totaling around 11.1 million in the US [2], and cancer survivorship is becoming more and more an important clinical topic [3]. Among various aspects of this survivorship, screening for second primary cancer (SPC) is an important topic. Cancer survivors are at higher risk to develop cancer [4,5], and SPC is associated with increased mortality [6]. Therefore, early detection by screening for SPC may be an effective way to lower the mortality of cancer survivors as a whole.

Previous studies show that cancer survivors are more likely to undergo cancer screening compared to people without cancer [7-9], nonetheless, the rate was shown to be suboptimal [10]. Some factors [10-12] have been shown to be associated with screening behaviors in cancer survivors, including knowledge and risk perception regarding SPC. However, these factors are rather an array of conceptual and subjective information of a patient that are not always clearly assessable by doctors in a typical clinical setting. In this situation, simpler clinical signs or indicators, if any, will be useful to identify who is at risk of not completing appropriate screening for SPC.

In this study, we investigated whether the mode of primary cancer detection (i.e. screen-detected vs. non-screen-detected) is associated with subsequent completion of all appropriate second primary cancer (SPC) screening in cancer survivors. We also investigated factors associated to the mode of primary cancer detection in order to evaluate other possible indicators that may be involved in the screening behavior of cancer survivors.

## Methods

### Participants and procedures

This study was performed as a part of an annual national survey to investigate the experience of cancer survivors. This study was approved by the Institutional Review Board of the National Cancer Center in Korea.

Using the quota sampling method, patients were recruited from 10 cancer centers (one national cancer center and the regional cancer centers in each of the nine Korean provinces) in Korea so that the perspective of patients with cancer common to Koreans, as well as that of patients of different gender and ages was represented as fairly as possible. Patients were included in this study if they were older than 18 years of age, used the inpatient or outpatient facilities of at least one of these 10 cancer centers, and agreed to participate. About 200 patients were recruited from each of the 10 cancer centers. To reflect national prevalence of each cancer types, 80% of the recruited patients were to be of the six major types of cancer (stomach, lung, liver, colon and rectal, breast, and cervical) and 20% of others.

Pilot surveys in each cancer center were first conducted using the survey methods employed in this study. No problems were found in the pilot study with patient understanding of the questions or with face or content validity of the questionnaires. Over a period of two months, cancer patients who gave written informed consent to participate in the study were interviewed by trained evaluators. A total of 1,956 cancer patients from the 10 cancer centers completed the interview process. In addition to the survey, medical chart audits were performed to obtain histological and Surveillance Epidemiology and End Results (SEER) stage information (version 2000) [13].

**Figure 1:**
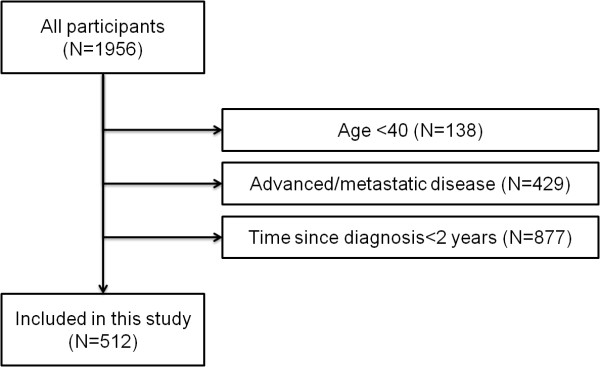
Sample selection algorithm for analysis used in this study.

For our study purposes in this particular study, from the original total of 1,956 cancer patients, we excluded patients younger than age 40 (N = 138), in order to specifically analyze the subpopulation of patients recommended to be screened regularly by the current guidelines in Korea (details of the guidelines described in the “Measures and outcomes” section below). Patients with advanced disease, namely those diagnosed with recurred or distant disease in respect to SEER staging, were excluded (N = 429), because the benefit of screening in those patients is limited due to their low 5-year survival rates. Patients with time since diagnosis less than 2 years were also excluded (N = 877) because in our outcome variable, completion of appropriate screening, screening is recommended at least every 2 years, and screening tests should be performed after cancer diagnosis. Of the original 1,956 subjects, 1,444 subjects were excluded and the final number of subjects for analysis was 512 (Figure 1).

### Measures and outcomes

The mode of detection of the cancer survivors’ first primary cancer, which is our main explanatory variable of interest, was determined by a survey question of “How was your cancer discovered?”, for which the answer choices were: (1) “I had a certain symptom of discomfort that prompted me to visit the hospital”; (2) “My cancer was discovered incidentally through routine screening”; (3) “My cancer was discovered incidentally while being tested for another condition”; and (4) “Others.” We defined “screen-detected” cancer patients as those who answered this question as (2) “My cancer was discovered incidentally through routine screening,” and defined “non-screen-detected” cancer patients as those who answered otherwise.

Questions regarding screening practices were adopted from the Korean National Health and Nutrition Survey (KNHANES) [14], and addressed whether individuals had ever undergone examinations for breast cancer (mammogram or breast sonography), stomach cancer (endoscopy or upper gastrointestinal series), cervical cancer (Papanicolaou test), or colorectal cancer (fecal occult blood test, sigmoidoscopy, colonoscopy, or barium enema). A positive answer to any screening question was followed by questions about the timing of the most recent examination (<1 year, 1–2 years, 2–5 years, >5 years, or none). We used “completion of all appropriate screening” as the main study outcome variable. Because to our knowledge there is no consensus regarding the optimal cancer screening strategy in Korean cancer survivors, an operational definition of appropriate screening in the current study was developed based on the National Cancer Screening Program in Korea [15]: (1) endoscopy or upper gastrointestinal series in the previous 2 years for stomach cancer screening (age ≥ 40); (2) sigmoidoscopy, colonoscopy, or barium enema in the previous 5 years for colorectal cancer screening (age ≥ 50); (3) mammogram or breast sonography in the previous 2 years for breast cancer screening (age ≥ 40); (4) Papanicolaou test in the previous 2 years for cervical cancer screening (age ≥ 30). Moreover, cancer survivors with specific first primary cancer that the screening aimed to detect were excluded from each calculation [9] (e.g. gastric cancer screening for gastric cancer patients were discarded), because such follow-up exams are carried out to monitor recurrence, rather than screen for SPC.

The survey also included socio-demographic factors known to be associated with screening practices, including age [16,17], gender [18], marital status [16,19], education [16,17,20], monthly household income [19,21,22], smoking status [17], and alcohol consumption [22]. Medical factors included type of cancer, SEER stage, comorbidity, and time since diagnosis. Information regarding the presence of comorbidities was also collected because such conditions are associated with cancer screening practices [20,23] and included hypertension, dyslipidemia, diabetes, osteoarthritis, rheumatoid arthritis, and cerebrovascular, cardiovascular, chronic liver, lung, kidney, or gastrointestinal diseases. Clinical variables, including the date of the primary cancer diagnosis, and stage of disease at the time of diagnosis were collected through review of medical records.

### Statistical analyses

Descriptive statistics were used to report screening practices of cancer survivors. We developed two multivariate logistic regression models: one to examine the factors associated with the completion of all appropriate screening, and the other to examine the factors associated with the mode of detection. Missing data were <1% for all variables. All analyses were conducted using STATA version 11.0. Statistical significance was specified when p-values were <0.05.

## Results

### Study population

The mean age of the study subjects was 59.6 ± 10.2 years; 265 (51.8%) were 60 years of age or older, and 250 (48.8%) were male. Stomach cancer was the most common diagnosis, followed by breast and colorectal cancer. Mean time since cancer diagnosis was 5.1 ± 3.3 years (Table 1).

**Table 1:** Characteristics of study participants

**Socio-demographic characteristics**	**N (%)**	**Medical characteristics**	**N (%)**
Age [Mean ± SD]	[59.6 ± 10.2 y]	Cancer types	
40 ≤ y < 60	247 (48.2)	Stomach	106 (20.7)
≥ 60 y	265 (51.8)	Lung	39 (7.6)
Gender		Liver	42 (8.2)
Female	262 (51.2)	Colon/Rectum	74 (14.5)
Male	250 (48.8)	Breast	113 (22.1)
Marital status		Cervix	14 (2.7)
Not married (single, divorced, widowed)	87 (17.0)	Others	124 (24.2)
Married	425 (83.0)	Stage	
Education		In situ & local	282 (55.1)
Less than high school	165 (32.2)	Regional	230 (44.9)
High school and above	347 (67.8)	Comorbidity	
Monthly household income		No	189 (36.9)
<2 million won	361 (70.5)	Yes	323 (63.1)
≥2 million won	151 (29.5)	Years since diagnosis [Mean±SD]	[5.1 ± 3.3 y]
Insurance		2 ≤ y <5	327 (63.8)
Medicaid/none/others	94 (18.4)	≥5 y	185 (36.1)
National health insurance	418 (81.6)		

Abbreviations: *SD* standard deviation, *N* number, *y* year.

### SPC screening practices

The overall SPC screening rate with the age and time corresponding to the guidelines of each cancer for stomach, colorectal, breast, and cervical cancer were 37.9%, 39.2%, 29.0%, 53.4%, respectively (Table 2). Completion rate of all appropriate SPC screening was 36.9% (Table 2) for overall, 32.0% for non-screen detected, and 50.4% for screen-detected survivors, respectively (Table 3).

**Table 2:** Screening practice for second primary cancer among cancer survivors

	Stomach Cancer Survivors*		**Lung Cancer Survivors**		**Liver Cancer Survivors**		Colorectal Cancer Survivors*		Breast Cancer Survivors*	Cervical Cancer Survivors*	**Other Cancer Survivors**		**All Survivors**
	**N (%)**		**N (%)**		**N (%)**		**N (%)**		**N (%)**	**N (%)**	**N (%)**		**N (%)**
	**Men**	**Women**	**Men**	**Women**	**Men**	**Women**	**Men**	**Women**	**N = 113**	**N = 14**	**Men**	**Women**	**N = 512**
	**N = 81**	**N = 25**	**N = 25**	**N = 14**	**N = 30**	**N = 12**	**N = 51**	**N = 23**			**N = 63**	**N = 61**	
Stomach cancer screening	NA	NA	16 (64.0)	12 (85.7)	12 (40.0)	5 (41.7)	19 (37.3)	8 (34.8)	55 (48.7)	5 (35.7)	30 (47.6)	32 (52.5)	194 (37.9)
Colorectal cancer screening**	32 (47.1)	8 (47.1)	13 (54.2)	9 (69.2)	16 (47.5)	3 (25.0)	NA	NA	32 (43.8)	5 (38.5)	23 (39.7)	23 (51.1)	164 (39.2)
Breast cancer screening***	NA	12 (48.0)	NA	8 (57.1)	NA	6 (50.0)	NA	8 (34.8)	NA	6 (42.9)	NA	36 (59.0)	76 (29.0)
Cervical cancer screening***	NA	14 (56.0)	NA	9 (64.3)	NA	5 (41.7)	NA	9 (39.1)	69 (61.1)	NA	NA	34 (55.7)	140 (53.4)
Complete cancer screening	45 (55.6)	9 (36.0)	14 (56.0)	6 (42.9)	10 (33.3)	3 (25.0)	19 (37.3)	6 (26.1)	27 (23.9)	4 (28.6)	21 (33.3)	25 (41.0)	189 (36.9)

Abbreviations: *NA* not applicable, *N* number.

*Only patients not having the specific type of cancer were included for the cancer screening, e.g. stomach cancer survivors were not subject to stomach cancer screening, etc.

**The overall rate of colorectal cancer screening has been calculated only among subjects of age 50 or more (N = 418). Cancer survivors who were younger than 50 were automatically classified as having completed colorectal cancer screening, because they were not recommended to be screened for colorectal cancer by current guidelines. Hence, male stomach cancer survivors younger than 50 were automatically classified as having completed all appropriate SPC screening without having received any screening exam.

***The overall rates of breast cancer screening and cervical cancer screening have been calculated only among female subjects (N = 262).

**Table 3:** Factors associated with completion of all appropriate SPC screening

	**Completion of All Appropriate SPC Screening**			
	**No**	**Yes**	**Univariate**	Multivariate*
	**N (%)**	**N (%)**	**OR (95% CI)**	**OR (95% CI)**
Age				
Old (≥60 y)	182 (68.7)	83 (31.3)	1.00	1.00
Young (40 ≤ y <60)	141 (57.1)	106 (42.9)	1.65 (1.15–2.37)	2.09 (1.32–3.31)
Gender				
Female	182 (69.5)	80 (30.5)	1.00	1.00
Male	141 (56.4)	109 (43.6)	1.76 (1.22–2.53)	1.45 (0.87–2.41)
Marital status				
Not married (single, divorced, widowed)	56 (64.4)	31 (35.6)	1.00	1.00
Married	267 (62.8)	158 (37.2)	1.07 (0.66–1.73)	0.69 (0.40–1.21)
Education				
Less than high school	120 (72.7)	45 (27.3)	1.00	1.00
High school and above	203 (58.5)	144 (41.5)	1.89 (1.26–2.83)	1.57 (0.96–2.57)
Monthly household income				
<2 million won	233 (65.5)	128 (35.5)	1.00	1.00
≥2 million won	90 (59.6)	61 (40.4)	1.23 (0.84–1.82)	0.91 (0.57–1.44)
Insurance				
Medicaid/none/others	65 (69.2)	29 (30.9)	1.00	1.00
National health insurance	258 (61.7)	160 (38.3)	1.39 (0.86–2.25)	1.46 (0.84–2.52)
Cancer types				
Stomach	52 (49.1)	54 (50.9)	1.00	1.00
Lung	19 (48.7)	20 (51.3)	1.01 (0.49–2.11)	1.46 (0.65–3.27)
Liver	29 (69.1)	13 (31.0)	0.43 (0.20–0.92)	0.47 (0.21–1.05)
Colon/Rectum	49 (66.2)	25 (33.8)	0.49 (0.27–0.91)	0.57 (0.29–1.12)
Breast	86 (76.1)	27 (23.9)	0.30 (0.17–0.54)	0.31 (0.15–0.64)
Cervix	10 (71.4)	4 (28.6)	0.39 (0.11–1.31)	0.65 (0.17–2.48)
Others	78 (62.9)	46 (37.1)	0.67 (0.34–0.96)	0.72 (0.40–1.28)
Stage				
In situ & local	182 (64.5)	100 (35.5)	1.00	1.00
Regional	141 (61.3)	89 (38.7)	1.15 (0.80–1.65)	1.19 (0.79–1.79)
Years since diagnosis				
2 ≤ y <5	200 (61.2)	127 (38.8)	1.00	1.00
≥5 y	123 (66.5)	62 (33.5)	0.79 (0.54–1.16)	0.95 (0.63–1.43)
Comorbidity				
No	124 (65.6)	65 (34.4)	1.00	1.00
Yes	199 (61.6)	124 (38.4)	1.19 (0.82–1.73)	0.94 (0.62–1.42)
Smoking, current				
No	305 (63.4)	176 (36.6)	1.00	1.00
Yes	18 (58.1)	13 (41.9)	1.25 (0.60–2.62)	1.04 (0.46–2.36)
Drinking, current				
No	288 (65.2)	154 (34.8)	1.00	1.00
Yes	35 (50.0)	35 (50.0)	1.87 (1.13–3.12)	1.53 (0.86–2.70)
Mode of first primary cancer detection				
Non-screen-detected	255 (68.0)	120 (32.0)	1.00	1.00
Screen-detected	68 (49.6)	69 (50.4)	2.16 (1.45–3.21)	2.13 (1.36–3.33)

Abbreviations: *N* number, *y* year, *OR* odds ratio, *CI* confidence interval.

*Adjusted for all variables in univariate analysis.

### Factors associated with completion of all SPC screening

In univariate analysis, younger age (odds ratio [OR], 1.65; 95% confidence interval [95% CI], 1.15–2.37), male gender (OR, 1.76; 95% CI, 1.22–2.53), higher education (OR, 1.89; 95% CI, 1.26–2.83), alcohol consumption (OR, 1.87; 95% CI, 1.13–3.12), and being screen-detected for primary cancer (OR, 2.16; 95% CI, 1.45–3.21) were associated with completion of all appropriate screening. In munltivariate-adjusted analysis, younger age (adjusted odds ratio [aOR], 2.09; 95% 95% CI, 1.32–3.31), and being screen-detected (aOR, 2.13; CI, 1.36–3.33) were found to be significantly associated with completion of all appropriate screening. There was also marginal significance with higher education (aOR, 1.57; CI, 0.96–2.57) (Table 3).

### Characteristics of patients by mode of first primary cancer detection

Screen-detected cancer survivors were significantly more likely to have higher household income (aOR, 2.23; CI, 1.39–3.58), have other comorbidities (aOR, 2.05; CI, 1.29–3.28). Survivors with 5 years or more since diagnosis were less likely to be screen-detected (aOR, 0.60; CI, 0.38–0.94). Prevalence of screen-detected patients were highest among stomach cancer patients, followed by liver, lung, breast, cervical, other, and colorectal cancer patients (36.8%, 33.3%, 30.8%, 29.2%, 21.4%, 19.4%, 16.2%) (Table 4).

**Table 4:** Characteristics of subjects by mode of first primary cancer detection

	**Mode of First Primary Cancer Detection**			
	**Non-screen-detected**	**Screen-detected**	**Univariate**	Multivariate*
	**N (%)**	**N (%)**	**OR (95% CI)**	**aOR (95% CI)**
Age				
Old (≥60 y)	201 (75.9)	64 (24.2)	1.00	1.00
Young (40 ≤ y <60)	174 (70.5)	73 (29.6)	1.32 (0.89–1.95)	0.91 (0.55–1.51)
Gender				
Female	192 (73.3)	70 (26.7)	1.00	1.00
Male	183 (73.2)	67 (26.8)	1.00 (0.68–1.49)	0.99 (0.56–1.74)
Marital status				
Not married (single, divorced, widowed)	67 (77.0)	20 (23.0)	1.00	1.00
Married	308 (72.5)	117 (27.5)	1.27 (0.74–2.19)	0.97 (0.53–1.79)
Education				
Less than high school	131 (79.4)	34 (20.6)	1.00	1.00
High school and above	244 (70.3)	103 (29.7)	1.68 (1.07–2.62)	1.41 (0.83–2.41)
Monthly household income				
<2 million won	284 (78.7)	77 (21.3)	1.00	1.00
≥2 million won	91 (60.3)	60 (39.7)	2.43 (1.61–3.67)	2.23 (1.39–3.58)
Insurance				
Medicaid/none/others	74 (78.7)	20 (21.28)	1.00	1.00
National health insurance	301 (72.0)	117 (28.0)	1.40 (0.82–2.40)	1.17 (0.64–2.15)
Cancer types				
Stomach	67 (63.2)	39 (36.8)	1.00	1.00
Lung	27 (69.2)	12 (30.8)	0.76 (0.35–1.68)	0.80 (0.34–1.88)
Liver	28 (66.7)	14 (33.3)	0.86 (0.40–1.82)	0.85 (0.38–1.90)
Colon/Rectum	62 (83.8)	12 (16.2)	0.33 (0.16–0.69)	0.33 (0.15–0.72)
Breast	80 (70.8)	33 (29.2)	0.71 (0.40–1.25)	0.56 (0.27–1.17)
Cervix	11 (78.6)	3 (21.4)	0.47 (0.12–1.78)	0.41 (0.09–1.77)
Others	100 (80.7)	24 (19.4)	0.41 (0.23–0.75)	0.38 (0.20–0.72)
Stage				
In situ & local	203 (72.0)	79 (28.0)	1.00	1.00
Regional	172 (74.8)	58 (25.2)	0.87 (0.58–1.29)	0.92 (0.59–1.42)
Years since diagnosis				
2 ≤ y <5	231 (70.6)	96 (29.4)	1.00	1.00
≥5 y	144 (77.8)	41 (22.2)	0.69 (0.45–1.04)	0.60 (0.38–0.94)
Comorbidity				
No	154 (81.5)	35 (18.5)	1.00	1.00
Yes	221 (68.4)	102 (31.6)	2.03 (1.31–3.14)	2.05 (1.29–3.28)
Smoking, current				
No	350 (72.8)	131 (27.2)	1.00	1.00
Yes	25 (80.7)	6 (19.4)	0.64 (0.26–1.60)	0.68 (0.26–1.80)
Drinking, current				
No	322 (72.9)	120 (27.2)	1.00	1.00
Yes	53 (75.7)	17 (24.3)	0.86 (0.48–1.55)	0.62 (0.32–1.20)

Abbreviations: *N* number, *y* year, *OR* odds ratio, *CI* confidence interval.

*Adjusted for all variables in univariate analysis.

## Discussion

To our knowledge, the current study, carried out with a relatively large nationally representative sample, is the first to examine the association between the mode of primary cancer detection and subsequent SPC screening in cancer survivors. We have shown that the mode of primary cancer detection may be used as a useful clinical indicator for SPC screening practices in cancer survivors. No specific comparison to other related studies for our key finding was possible because our study is the only study that investigated the association between the mode of primary cancer screening and subsequent SPC screening in cancer survivors, which is a unique setting different from primary cancer screening.

In our study, only 36.9% of cancer survivors completed all age, sex- appropriate screening for SPC. This figure is similar to previous studies performed in Korea [12], and confirms the need to increase screening rates in this population. Therefore, identification of high risk group for non-completion of SPC screening would be meaningful from both clinical and public health care perspectives.

Our results show that screen-detected cancer survivors are approximately twice more likely to receive all appropriate SPC screening, even after controlling for other covariates which may affect cancer screening behaviors. Therefore, the mode of primary cancer detection separates two subpopulations of cancer survivors with differing risk for not receiving proper SPC screening, serving as a clinical indicator.

According to the Health Belief Model (HBM), people will take action to prevent, screen for, or control their health conditions if they believe they are susceptible, believe the condition would have serious consequences, and believe there is benefit to taking a course of action [24]. The role of better knowledge [25,26], a positive attitude [27], and perceived risk [28] in cancer screening practices are well known in the general population.

Similar findings have been reported in cancer survivors regarding SPC screening about the role of knowledge [12] and risk perception of SPC [11,12]. It was reported that cancer survivors often could not differentiate SPC from “recurrence” or “metastasis,” and could not make a distinction between “cancer screening” and “routine surveillance test” after cancer treatment [29]. Such lack of knowledge [12] was significantly associated with failed completion of all appropriate SPC screening in cancer survivors. In addition, a considerable portion of cancer survivors perceived their cancer risk as lower than that of the general population, and such misperception of SPC risk were negatively associated with screening for SPC. While those studies provide theoretical insight on the screening behavior of cancer survivors, it is impractical to collect information regarding their knowledge and risk perception in the clinical setting, their clinical utility being limited.

We suspect that the mode of primary cancer detection could be an indicator of such knowledge or risk perception of SPC in cancer survivors for the following reasons. First, people who generally have good knowledge on the benefit of cancer screening and proper risk perception are more likely to receive cancer screening, and in case they are diagnosed with cancer, they are most likely to become screen-detected cancer survivors. Second, the personal experience of discovering their cancer through screening would provide the knowledge that screening is critical to early detection and effective treatment of cancer. Unfortunately, our survey did not include specific questions about the cancer survivors’ knowledge and risk perception, therefore, our explanation for the association can only remain hypothetical.

It is interesting to note one of our observations that having been diagnosed with primary cancer for more than 5 years is associated with being non-screen-detected (Table 4). This can be attributed to the recently developed and propagated national cancer screening program in Korea which, launched in 1999, has successfully increased its participation rate from 12.7% in 2002 to 27.8% in 2008, and is projected to have increased further [30]. As our survey has been carried out in 2008, our observations reflect such drastic changes in national screening rates.

The rate of liver or lung cancer survivors to be screen-detected has been shown to be overall higher than that of stomach, colorectal, breast, or cervical cancer survivors, which are cancer types with specific screening recommendations (Table 4). This may be interpreted as counterintuitive and we have two main explanations for these observations. One, considering our study excluded subjects with less than 2 years since diagnosis, recurrence, or distant disease, and also considering the relatively high 5-year mortality rate of lung or liver cancer in Korea [31], we suspect there is a selection bias toward screen-detected lung or liver cancer survivors. Two, early detection of lung or liver cancer involves low dose chest computed tomography or abdomen ultrasonography, respectively, which are procedures in Korea only offered (to asymptomatic subjects without particular indications) in commercial private health screening programs, but not in government screening programs that strictly abide to specific screening recommendations [32]. Therefore, mostly health-conscious subjects are exclusively willing to pay the high price for such health packages, a phenomenon not unique in Korea [33]. This would be a source of selection bias not exclusive to our study, but to lung or liver cancer survivors (with more years since diagnosis) in general, that well explains such aforementioned counterintuitive observations. We regard these observations to be very interesting, because a further study on the particular nature of mode of primary cancer detection (whether it be screening via government programs or commercial programs) and its association to completion of all appropriate SPC screening will most likely add predictive value to identifying high risk cancer survivors at risk of not properly receiving proper SPC screening.

It is to be emphasized that our results and implications are especially significant in that we considered completion of all appropriate SPC screening rather than single type SPC screening. Most previous studies investigating the barriers associated to screening have evaluated screening of single, specific cancer types [17,22], and according to a large US study, only 3% of women and 5% of men older than 50 had completed all appropriate cancer screening for their age and sex [34]. This fact should be noted in context to 1) various public campaigns for single type cancer screening, as in the case of pink ribbon breast cancer campaign [35], and 2) many practices that provide only one or two screening tests. In fact, in our results, compared to stomach cancer survivors, breast cancer survivors were significantly associated to non-completion of all appropriate SPC screening (aOR 0.31, 95% CI 0.15–0.64; Table 3). We therefore assert that analysis of factors associated with completion of all appropriate SPC screening would more accurately reflect cancer survivors’ knowledge and perception of SPC screening, and hence be better predictive of their SPC screening behavior, than that of single type SPC screening.

There are several limitations to our current study. First, there is a source of imprecision in terms of the definition of SPC screening in our sample and its correspondence to time since diagnosis, especially for colorectal cancer screening. Due to limitation of sample size, our analysis includes cancer survivors with time since diagnosis from 2 years and more. Because colorectal cancer screening guidelines recommend screening every 5 years after age 50, those with time since diagnosis less than 5 years (and older than 50) may not have been subject to SPC screening for colorectal cancer and consequently we may have accounted for primary cancer screening. However, sensitivity analysis (data not shown) among patients with time since diagnosis beyond 5 years show similar magnitude of association and statistical significance of factors associated with completion of all appropriate SPC screening. Second, the assessment of cancer screening practices was based on participants’ self-report, subject to recall bias. Although we used carefully phrased questions which were used in KNHANES [14] to maximize accuracy, it is possible that survivors with less knowledge may have also had more difficulty understanding the questions correctly or may have undergone screening tests without realizing that they were performed for screening purposes. Third, despite of our study’s multicenter, nationwide design, our study sample was not large enough to allow subgroup analysis by primary cancer type. It is possible that the behaviors may differ among survivors with different primary cancers. Fourth, we cannot statistically assess the representativeness of our sample. Instead, we employed quota sampling to obtain a similar distribution of cancer types to the general Korean cancer population. In addition, our gender and age group distributions were not biased. Overall, we assert that this limitation does not lead to serious problems with internal validity or representation, and there has been previous studies based on the same survey [36].

Despite above-mentioned limitations, our study suggests that the mode of primary cancer detection, a readily available clinical information, can be used as an indicator for screening practice for SPC in cancer survivors. Education about the importance of SPC screening will be helpful particularly for cancer survivors whose primary cancer was not screen-detected.

## Conclusions

We have identified non-screen-detected cancer survivors as a subpopulation at risk of improper SPC screening behavior. We suggest that all cancer survivors must be inquired about how their primary cancer were detected, and extensive emphasis and education on the importance of SPC screening must be provided, especially, for non-screen-detected cancer survivors by their physicians in the clinic.

## Competing interests

All authors have no potential financial, professional or personal conflicts by publishing this manuscript. J.-H. Park was supported by a research grant from National Cancer Center Grant No. 0910191 & 1210151, Korea. This government organization had no influence on any aspect relevant to this study.

## Authors’ contributions

DWS and JHP conceived of the study, participated in design and coordination of the study. DWS, JHP, WYC, SPL, CYY were involved in acquisition of data. BS performed the statistical analysis and drafted the manuscript. DWS, JHP, SYK, BLC, ECP gave administrative support, and revised the manuscript. All authors read and approved the final manuscript.

## Pre-publication history

The pre-publication history for this paper can be accessed here:

http://www.biomedcentral.com/1471-2407/12/557/prepub
